# Evaluation of Diagnostic and Triage Accuracy and Usability of a Symptom Checker in an Emergency Department: Observational Study

**DOI:** 10.2196/38364

**Published:** 2022-09-19

**Authors:** Hamish S F Fraser, Gregory Cohan, Christopher Koehler, Jared Anderson, Alexis Lawrence, John Pateña, Ian Bacher, Megan L Ranney

**Affiliations:** 1 Brown Center for Biomedical Informatics Warren Alpert Medical School Brown University Providence, RI United States; 2 School of Public Health Brown University Providence, RI United States; 3 Warren Alpert Medical School Brown University Providence, RI United States; 4 Department of Emergency Medicine Brown University Providence, RI United States; 5 Harvard Medical Faculty Physicians Department of Emergency Medicine St Luke's Hospital New Bedford, MA United States; 6 Brown-Lifespan Center for Digital Health Providence, RI United States

**Keywords:** mobile health, mHealth, symptom checker, diagnosis, user experience

## Abstract

**Background:**

Symptom checkers are clinical decision support apps for patients, used by tens of millions of people annually. They are designed to provide diagnostic and triage advice and assist users in seeking the appropriate level of care. Little evidence is available regarding their diagnostic and triage accuracy with direct use by patients for urgent conditions.

**Objective:**

The aim of this study is to determine the diagnostic and triage accuracy and usability of a symptom checker in use by patients presenting to an emergency department (ED).

**Methods:**

We recruited a convenience sample of English-speaking patients presenting for care in an urban ED. Each consenting patient used a leading symptom checker from Ada Health before the ED evaluation. Diagnostic accuracy was evaluated by comparing the symptom checker’s diagnoses and those of 3 independent emergency physicians viewing the patient-entered symptom data, with the final diagnoses from the ED evaluation. The Ada diagnoses and triage were also critiqued by the independent physicians. The patients completed a usability survey based on the Technology Acceptance Model.

**Results:**

A total of 40 (80%) of the 50 participants approached completed the symptom checker assessment and usability survey. Their mean age was 39.3 (SD 15.9; range 18-76) years, and they were 65% (26/40) female, 68% (27/40) White, 48% (19/40) Hispanic or Latino, and 13% (5/40) Black or African American. Some cases had missing data or a lack of a clear ED diagnosis; 75% (30/40) were included in the analysis of diagnosis, and 93% (37/40) for triage. The sensitivity for at least one of the final ED diagnoses by Ada (based on its top 5 diagnoses) was 70% (95% CI 54%-86%), close to the mean sensitivity for the 3 physicians (on their top 3 diagnoses) of 68.9%. The physicians rated the Ada triage decisions as 62% (23/37) *fully agree* and 24% (9/37) *safe but too cautious*. It was rated as *unsafe and too risky* in 22% (8/37) of cases by at least one physician, in 14% (5/37) of cases by at least two physicians, and in 5% (2/37) of cases by all 3 physicians. Usability was rated highly; participants *agreed* or *strongly agreed* with the 7 Technology Acceptance Model usability questions with a mean score of 84.6%, although “satisfaction” and “enjoyment” were rated low.

**Conclusions:**

This study provides preliminary evidence that a symptom checker can provide acceptable usability and diagnostic accuracy for patients with various urgent conditions. A total of 14% (5/37) of symptom checker triage recommendations were deemed unsafe and too risky by at least two physicians based on the symptoms recorded, similar to the results of studies on telephone and nurse triage. Larger studies are needed of diagnosis and triage performance with direct patient use in different clinical environments.

## Introduction

### Background

Improving medical diagnosis is a high priority, with evidence that the average American will experience at least one important misdiagnosis in their lifetime and that 5% of outpatient diagnoses are incorrect [1]. Although a number of initiatives have sought to improve outpatient diagnosis by physicians and other health care workers [2,3], less attention has been paid to the key role patients play in ensuring they receive effective and timely diagnosis and treatment [4]. Late recognition of many diseases can lead to poor outcomes, whether for acute diagnoses such as myocardial infarction and stroke or for more chronic diseases, including renal failure [5] and carcinomas [6]. Diagnostic and triage apps designed for patients, often called symptom checkers, have become widely available to the general public over the last decade [7]. Leading symptom checkers claim millions of users annually; for example, iTriage claimed 50 million users per year in 2015 [8], and WebMD claimed 4 million users per month in 2019 [9].

These apps typically require the user to enter limited demographic information followed by their chief complaint or symptom. They then ask follow-up questions on the symptoms, which vary in number and strategy by app [10,11]. The output is one or more diagnoses or a triage level and may include suggestions for actions that the user should take, including seeking routine or urgent care. Symptom checker apps differ from diagnostic tools for health care workers; in most cases, symptom checkers do not use data on vital signs, physical examination, current medications, or investigations [12,13].

The context of a person using a symptom checker app in a home or community setting can be similar to calling an urgent care helpline such as the NHS111 service in the United Kingdom [14] (although likely involving less urgent conditions). However, it does not include access to the patient’s care record or to human assistance in navigating the algorithm. App use is typically promoted by private companies that develop them; academic developers of apps [15]; health systems that have developed their own symptom checkers (eg, Mayo Clinic [16]); or health systems that have partnered with companies, such as Babylon with the National Health Service in the United Kingdom [17] and Ada with Sutter Health in the United States [18]. Symptom checkers have the potential to help patients identify the correct diagnosis for their problem and the appropriate action to take in seeking care. Symptom checkers could be particularly helpful in improving care for patients with limited access to health systems, such as in rural areas and other underserved communities worldwide. These apps could also assist people uncertain of the significance of symptoms with potentially serious underlying causes, such as chest pain or headache. Alternatively, a symptom checker might miss important diagnoses, discourage users from seeking urgent care, or overwhelm health systems with patients who have nonurgent problems. The latter issue was observed with the phone triage system NHS111 [19] and in a study of the use of telehealth consultations that increased patient contacts rather than just displacing in-person care [20]. Symptom checkers have seen extensive use during the COVID-19 pandemic, with evaluation studies showing good diagnostic accuracy for some systems but significant underdiagnosis in some nationally deployed COVID-19 symptom checker apps [21]. However, many patients with COVID-19 have few or no symptoms, limiting potential sensitivity in the absence of additional data such as pulse oximetry.

Despite ample business promotion of symptom checkers, little rigorous evidence supports their effectiveness, safety, accuracy, ability to decrease the load on health systems, or usability by the full range of users or patients [7,22]. Most studies to date have used clinical vignettes—patient histories with “correct” diagnoses created by physicians—to evaluate key metrics [12,13,23,24]. Although such studies have played an important role in identifying gaps in coverage or weaknesses in diagnostic algorithms, they do not reproduce the experience of patients using a symptom checker. In addition, the vignettes may be less challenging to diagnose than real patient histories collected in an emergency situation, as illustrated in a recent study that included vignettes created using actual patient presentations to an urgent health care hotline [13]. To date, studies that have been based on direct patient use of symptom checkers have enrolled few acute patients or had poorly defined study populations and outcomes [25,26]. As symptom checkers are designed to be used in a home or community setting without direct health care support, to address the critical question of patient safety, it is necessary to enroll patients with serious and potentially life-threatening diseases. For example, in a study of routine symptom checker use in a health system in California, 29% of assessments were for patients considered by the clinical team to be high urgency [18].

### Ada Health Symptom Checker

The Ada Health symptom checker’s diagnosis algorithm was developed with the original goal of assisting clinicians with the diagnosis of rare diseases. Since the launch of the symptom checker in 2016, its use has grown rapidly in Germany, the United Kingdom, and the United States. A total of 11 million users have carried out 23 million health assessments. It is available in 11 languages and, in 2020, it was rated as the most commonly used symptom checker in 150 countries [27]. On first use of the Ada app, the user is questioned about their age, sex, gender, and a limited number of pre-existing diseases. They are then asked for their chief complaint. A series of questions then follows in the manner of a “chatbot.” Upon completion of the question-and-answer phase, the user is given a list of 1 to 5 “condition suggestions” equivalent to a differential diagnosis and a recommendation for the level of urgency with which to seek care. The underlying algorithm is a Bayesian network. Previous studies of Ada have shown good levels of performance on a wide range of diagnoses using clinical vignettes, including a large study of 8 symptom checkers using 200 vignettes (led by a team from Ada Health with support in design and analysis from outside experts, including HF) [13] and a study by Ceney et al [10], which was independent from Ada. The choice of Ada for this study was based on its widespread use in many countries, preliminary evidence of strong performance, and a willingness to collaborate with outside, independent research teams, as shown by the wide range of published evaluation studies [13,28,29]. The authors also considered other symptom checkers, including conversations with YourMD Ltd, and have independently tested the Ada, Isabel, and WebMD symptom checkers on the diagnosis of chest pain [30].

The study was designed to recruit patients seeking urgent care in an emergency department (ED).

The research questions for this study were as follows: (1) Could patients presenting with an urgent clinical problem effectively record their symptoms and did they find Ada easy to use? (2) Did patient characteristics affect their successful use of the app? (3) What was the sensitivity of Ada for the diagnoses of the ED physicians who saw the patients? (4) Were the diagnoses suggested by Ada as sensitive to the ED physicians’ diagnoses as the diagnoses suggested by physicians using the same clinical data? (5) Were the diagnoses suggested by Ada considered reasonable by the physicians? (6) Were the triage suggestions from Ada considered reasonable and safe by the physicians? (7) Did access to vital sign data improve the diagnostic performance of the reviewing physicians?

## Methods

### Overview

The symptom checker was tested with direct use by patients coming to the Rhode Island Hospital (RIH) ED with a wide range of presenting complaints. The sample population was designed to include “patients with acute or serious medical conditions presenting to an emergency department.” Patients were eligible if they were English-speaking, aged ≥18 years, presenting for emergency evaluation of a medical (nontrauma and non–mental health) problem, and deemed by the triage nurse to not be critically ill (Emergency Severity Index score of 2-5). In addition, they had to be able to consent and complete the symptom checker assessment before physician evaluation.

Participants were approached after initial nurse triage but before physician assessment by a research assistant (CK) on a convenience sample of shifts. After obtaining consent from the participants, they were provided with an iPad with the study software installed and followed the symptom checker prompts. Upon completion of the symptom checker questions, they were then asked to complete a usability survey using REDCap (Research Electronic Data Capture; Vanderbilt University), a Health Insurance Portability and Accountability Act–compliant survey software [31]. The survey questions were of three types: (1) demographic data, including age, sex, race, ethnicity, and socioeconomic status; (2) six questions on the participants’ use of web-based resources and health information–seeking behavior; and (3) eleven questions from the Technology Acceptance Model [32]. Participants were compensated with US $20 for their time. The version of Ada used in the study used the same diagnostic algorithm as the production system but returned the results as a PDF file emailed to the study team; the patients did not see the results.

Symptom checker data were compiled into deidentified files. Each file included the patient’s answers to the symptom checker questions, up to five diagnoses from the symptom checker (termed “condition suggestions” by Ada), and a recommended triage action for the patient (termed “urgency advice” by the Ada app). An example is shown in Multimedia Appendix 1. No symptom checker data were seen by the patient or the physician caring for them. Subsequently, the discharge diagnoses, vital signs, physical examinations, and laboratory or other study results from the ED visit were abstracted from the electronic health record (EHR). If the ED discharge diagnoses were unclear, an attending ED physician independent of the patient’s care adjudicated.

To assess performance, 3 independent, board-certified emergency physicians reviewed the data summaries generated by the symptom checker, blinded to the ED diagnoses. REDCap was used to present the data to allow each physician to complete the following tasks in sequence: (1) review the patient-reported symptom checker symptom data and generate their own differential diagnosis and triage level without access to the Ada diagnosis or condition suggestion or the patient’s chart, (2) review the patient’s vital signs and then restate their top 3 diagnoses and triage levels, and (3) review and critique the Ada diagnoses and triage levels for the case. The results from the 3 ED physicians’ critiques of Ada were combined to create an assessment of the appropriateness of the diagnoses and triage levels by majority voting.

The 95% CIs were calculated using the proportions method, and the diagnostic accuracy results were compared using the chi-square test. Interrater agreement between the symptom checker, discharge diagnoses, and independent physician diagnoses was calculated by comparing their percentage of agreement on the ED diagnoses (ie, in what percentage of cases did 2 clinicians match the same ED diagnosis). Clinicians were then compared with Ada in the same fashion, limiting Ada to its top 3 diagnoses. The *comprehensiveness* and *relevance* metrics were calculated for Ada and the physicians to account for multiple diagnoses in each list. Comprehensiveness was calculated as the percentage of ED diagnoses matched by the differential diagnoses of Ada or the physicians (similar to sensitivity). Relevance was calculated as the percentage of the diagnostician’s (Ada or the physicians) diagnoses that matched the ED diagnoses (similar to the positive predictive value) [33]. Free-text comments were analyzed thematically by 2 authors (HSFF and GC), and differences were resolved through discussion. For the user survey, descriptive statistics were calculated for demographic and Likert-scale data using Microsoft Excel.

### Ethics Approval

The study was approved by the Interventional Review Board, Research Data Protection Office, Lifespan Healthcare, Providence, Rhode Island (1439681-3). Institutional review board approval was obtained before initiation of the study.

## Results

### Overview

**Figure 1:**
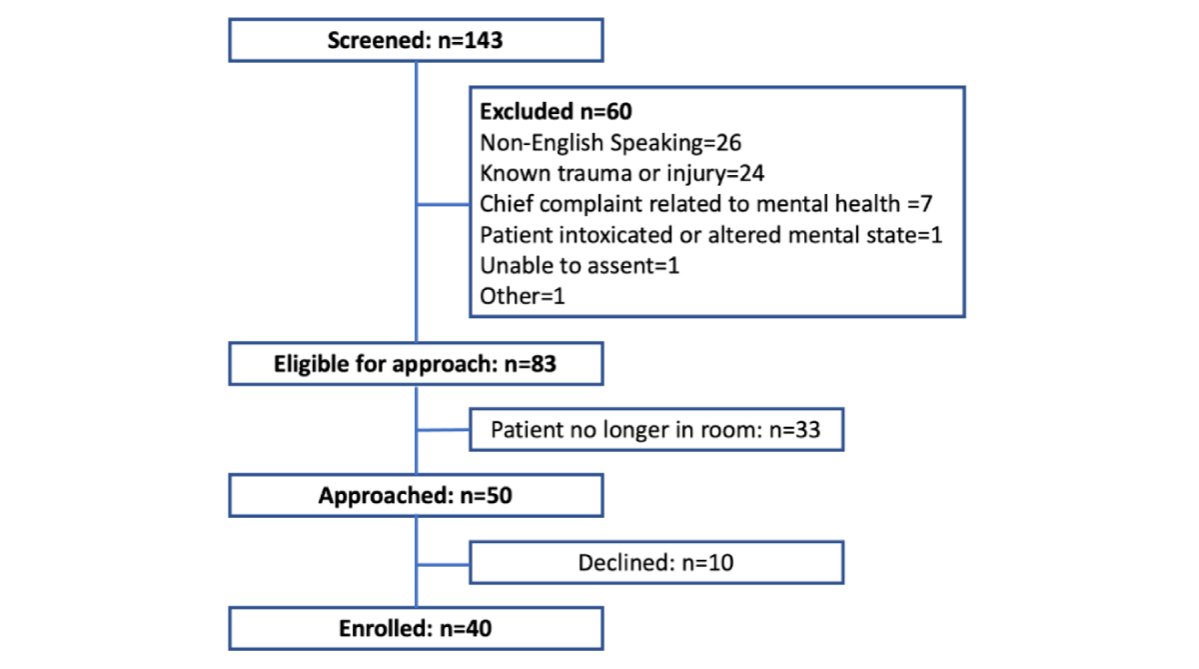
Patient recruitment and reasons for exclusion. Owing to technical problems, 37 cases were usable for analysis of triage, and 33 cases were usable for analysis of diagnoses.

Over 5 days in September 2019 to October 2019, a total of 143 patients presented to the ED and were screened, and 84 (58.7%) were potentially eligible. Of these 84 patients, 50 (60%) were approached, and 40 (48%) consented. Figure 1 shows the reasons for exclusion. Of the consenting participants, 65% (26/40) were women, with a mean age of 39.3 (SD 15.9; range 18-76) years. Table 1 shows the breakdown by education level and receipt of public assistance for the study and by race and ethnicity for the study and for all patients seen in the ED between September 2019 and October 2019. The study population had a higher proportion of female patients, had a younger mean age, and was more diverse, with a higher proportion who identified as Hispanic and more patients from less common racial and ethnic groups.

Of the 40 patients enrolled, 7 (18%) had incomplete data: 2 (29%) were missing an ED assessment as the patients left against advice, symptom checker assessment files were not generated by Ada in 3 (43%) cases, and Ada assessment files had symptom data and triage information but no diagnosis (with one of these also missing an ED assessment) in 3 (43%) cases. These problems were related to the research environment for Ada, not the symptom checker itself, and were resolved for a subsequent study in primary care. Therefore, overall, 83% (33/40) of the cases had both a full Ada assessment and an ED assessment.

**Figure 2:**
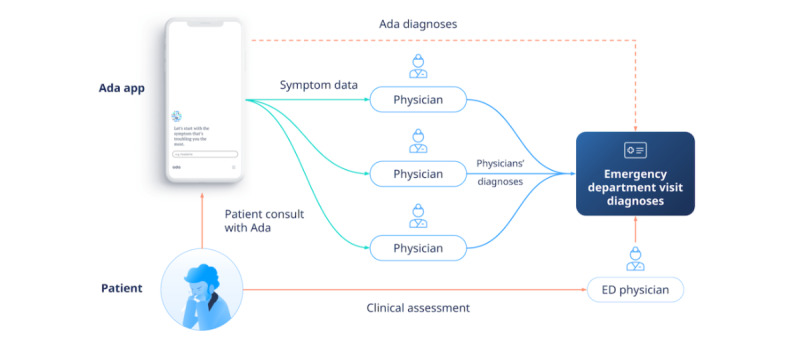
Primary comparison of the diagnoses from Ada and the physicians reviewing the Ada data with the emergency department (ED) physician diagnosis.

Of these 33 complete cases, 22 (67%) had a clear discharge diagnosis, and the other 11 (33%) had a symptom listed as the final diagnosis. Of these 11 cases, 6 (55%) had a diagnosis of “chest pain,” and 2 (18%) had a diagnosis of “back pain.” The other 3 cases had poorly specified symptoms: 2 (67%) of “abdominal pain” and 1 (33%) of “dizziness.” Consequently, diagnostic accuracy was measurable based on the ED assessment for 30 cases (clear ED diagnosis, myocardial infarction screen, or back pain). There was a mean of 2.5 diagnoses per case (range 1-6) based on the ED record. The review of diagnoses by the 3 independent physicians included 33 cases with complete data, and their review of triage accuracy included all 37 cases with triage data from Ada. The patients were seen in a major ED and level-1 trauma center. Considering the 33 cases, all 6 (18%) patients with chest pain were screened for acute myocardial infarction (AMI). Of the 6 patients, 1 (17%) was admitted with cardiac ischemia, 1 (3%) had a head injury and concussion—possible intercranial hemorrhage—and 1 (3%) had acute appendicitis. The details of the 40 cases, including presenting complaint, ED diagnoses, disposition, and if there was missing data, are provided in Multimedia Appendix 2. One of the reviewing physicians also acted as an expert opinion on uncertain ED diagnoses >6 months after reviewing the Ada data. Figure 2 shows the primary evaluation of the diagnoses from Ada and the physicians with the ED diagnosis.

**Table 1:** Breakdown by self-reported race, education level, and receipt of public assistance (some identified as 2 races). Data from the 16,708 general emergency department (ED) patients seen between September 2019 and October 2019 are shown for comparison. The category “other” in the ED data mapped 88% to “Hispanic or Latino” (N=40).

Characteristic		This study, n (%)	General ED (total patients=16,708), n (%)
**Race**			
	American Indian or Alaska Native	2 (5)	37 (0.22)
	Asian	3 (8)	238 (1.42)
	Native Hawaiian or other Pacific Islander	1 (3)	36 (0.22)
	Black or African American	5 (13)	2203 (13.19)
	White	27 (68)	9906 (59.29)
	Other	4 (10)	3886 (23.26)
	Prefer not to answer	1 (3)	51 (0.31)
Ethnicity—Identified as Hispanic or Latino		19 (48)	4084 (24.44)
**Education level**			
	Some high school	3 (8)	—^a^
	High school degree or equivalent (eg, GED^b^)	8 (20)	—
	Some college	10 (25)	—
	Trade or technical or vocational training	3 (8)	—
	Associate’s degree	5 (13)	—
	Bachelor’s degree	7 (18)	—
	Master’s degree	4 (10)	—
**Receipt of public assistance**			
	Yes	10 (25)	—
	No	28 (70)	—
	Preferred not to say	2 (5)	—

^a^Education level and receipt of public assistance was not recorded in general ED population.

^b^GED: General Educational Development.

### Metric 1: Comparing Ada Diagnoses With ED Diagnoses

Diagnostic accuracy was measured by the number of Ada diagnoses that matched at least one of the ED discharge diagnoses. For the sensitivity analysis, we considered whether one of the ED discharge diagnoses was Ada’s top 1 diagnosis, whether it was in the top 3, or whether it was in the top 5. The top 1 diagnosis matched in 30% (9/30; 95% CI 14%-46%) of cases, the top 3 diagnoses matched in 63% (19/30; 95% CI 46%-81%) of cases, and the top 5 diagnoses matched in 70% (21/30; 95% CI 54%-86%) of cases.

### Metric 2: Comparing Independent Physician Diagnoses With ED Diagnoses

The independent physicians were asked to provide up to 3 diagnoses after reviewing symptom checker data with and without vital signs but were blinded to Ada’s results. The mean percentage match between diagnoses for the 3 physicians was the top 1 match in 47% (14/30; 95% CI 36%-57%) of cases and the top 3 matches in 69% (20.7/30; 95% CI 59%-78%) of cases. For *physician 1*, the top diagnosis matched the ED diagnosis in 40% (12/30; 95% CI 23%-58%) of cases, and the top 3 diagnoses matched in 70% (21/30; 95% CI 54%-86%) of cases. For *physician 2*, the top diagnosis matched in 57% (17/30; 95% CI 39%-74%) of cases, and the top 3 diagnoses matched in 70% (21/30; 95% CI 54%-86%) of cases. For *physician 3*, the top diagnosis matched in 43% (13/30; 95% CI 26%-61%) of cases, and the top 3 diagnoses matched in 67% (20/30; 95% CI 50%-84%) of cases.

**Figure 3:**
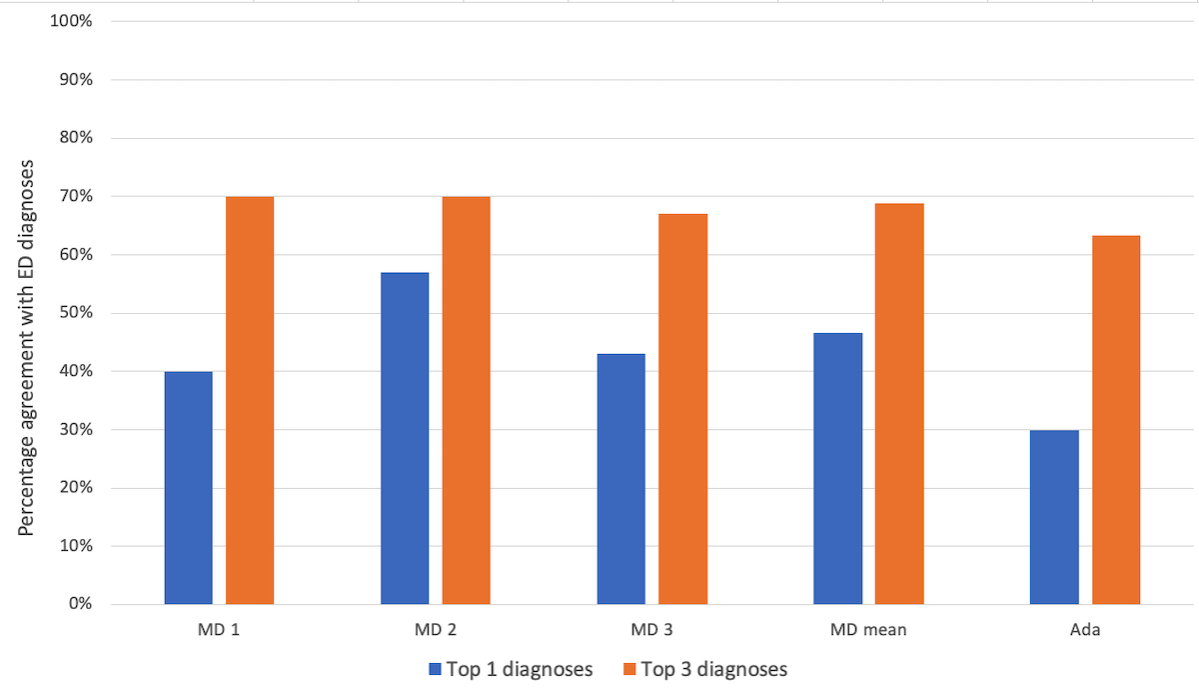
Percentage of cases with at least one match to the final emergency department (ED) diagnosis (MD=physician).

The comparison of the top 1 match for Ada (metric 1) with the combined top 1 matches for the 3 physicians (metric 2) was not significant (*P*=.07). The results of the top 1 matching diagnosis for *physician 2* showed a significantly higher performance than Ada (*P*=.02). Matching performance on the top 3 diagnoses was not significantly different between Ada and any of the physicians (Figure 3).

Table 2 shows the percentage of agreement among the pairs of clinicians and clinicians paired with Ada, showing the percentage of cases in which they matched the same ED diagnosis on their top 3 diagnoses. Overall, there was higher agreement between pairs of physicians than between physicians and Ada, but these differences were not statistically significant. The mean level of agreement for Ada was 57% if the top 5 diagnoses were included.

**Table 2:** Pairwise comparisons of percentage of agreement between physicians and with symptom checker diagnoses to assess interrater agreement (N=30).

Pair number	Pair	Agreement, n (%)
1	Physician 1-physician 2	19 (63)
2	Physician 1-physician 3	17 (57)
3	Physician 1-Ada	13 (43)
4	Physician 2-physician 3	17 (57)
5	Physician 2-Ada	17 (57)
6	Physician 3-Ada	15 (50)
7	Physician 1-ED^b^ diagnoses	20 (67)
8	Physician 2-ED diagnoses	21 (70)
9	Physician 3-ED diagnoses	22 (73)
10	Ada-ED diagnoses	19 (63)

^a^Ada mean (rows 3, 5, and 6): 15 (50%); physician mean (rows 1, 2, and 4): 18 (60%); overall mean: 53%.

^b^ED: emergency department.

### Metric 3: Evaluating Reasonableness of Ada Diagnoses and Triage Urgency Through Independent Physicians

There were a total of 130 Ada diagnoses; these were reviewed by the 3 clinicians. On the basis of agreement of at least two reviewers, the results were 39% (50/130) “very reasonable,” 30% (39/130) “reasonable,” 16.2% (21/130) “neither reasonable nor unreasonable,” 15% (19/130) “unreasonable,” and <1% (1/130) “very unreasonable.” The diagnosis considered very unreasonable was age-related farsightedness in a patient presenting with chest pain and headache. Other Ada diagnoses for this case were eye strain, hypertensive emergency, high blood pressure, and low blood sugar. See Table 3 for other examples and additional information on the symptoms entered and associated diagnoses.

**Table 3:** Diagnoses considered *very unreasonable* by a physician reviewer and *unreasonable* by one or more. The italicized diagnosis was rated as *very unreasonable* by 2 reviewers (additional symptoms were reported to Ada).

Conditions critiqued in Ada diagnoses	Chief complaint (ED^a^)	Additional information
Heatstroke	Dizziness, nausea, and headache	Other Ada diagnoses: low blood sugar and viral stomach bug, considered reasonable
*Age-related farsightedness*	Chest pain and headache	Symptoms entered into Ada included eye strain, reduced vision, laterality (both eyes), and no sudden onset
Hereditary angioedema	Left upper quadrant abdominal pain	Top Ada diagnosis: pancreatitis, considered reasonable or very reasonable
Hereditary angioedema, abdominal wall hematoma, or Meckel diverticulum	Abdominal pain	Top Ada diagnosis: appendicitis, matched by all 3 physicians

^a^ED: emergency department.

When the clinicians assessed Ada’s suggested triages, 62% (23/37) were rated as *fully agree*, 24% (9/37) were rated as *safe but too cautious*, and 14% (5/37) were rated as *unsafe and too risky*. A total of 22% (8/37) were rated as *unsafe and too risky* by at least one physician, but only 5% (2/37) were found so by 100% (3/3) of the physicians. Each case report generated by Ada had an overall urgency level; this was normally based on the diagnosis with the most urgent level of triage. However, 11% (4/37) cases had a more urgent triage level for diagnoses ranked at lower probabilities. Including those more urgent triage levels in the analysis reduces the rate of undertriage supported by at least two physicians to 5% (2/37) but does not change overtriage, increasing the *fully agree* category to 70% (26/37). The details of the reviews of triage and diagnoses in the *unsafe and too risky* category are shown in Multimedia Appendix 3.

### Metric 4: Comprehensiveness and Relevance Results

Compared with the ED diagnoses, the mean *comprehensiveness* for Ada’s top 3 diagnoses was 41%, and the mean *relevance* was 22%. For the top 5 diagnoses, they were 46% and 24%, respectively. Considering only the top 1 diagnosis, Ada’s *relevance* was 33%. The mean *comprehensiveness* for the physicians was 46% (range 41%-54%), and the mean *relevance* was 27.7% (range 25%-32%). The mean *relevance* for the physicians’ top 1 diagnosis was 47.8% (range 40%-60%).

### User Survey

All 40 participants successfully completed the user survey (and were not given access to the Ada results). The results of the survey are shown in Tables 4 and 5. Overall, they reported a high level of use of cell phones to send SMS text messages and enter and view data; however, only 53% (21/40) used a computer at work. When seeking medical advice, the most frequent source was doctors (physicians; 22/40, 55%), with web-based sources being second at 40% (16/40). A total of 83% (33/40) searched for medical symptoms on the web at least sometimes, and 28% (11/40) had previously used a symptom checker. Regarding overall satisfaction, participants were evenly split among *satisfied, neutral*, and *unsatisfied*. Only 23% (9/40) said that the use of Ada was “enjoyable”; however, 70% (28/40) were *likely* or *very likely* to recommend it to a friend.

Participants *agreed* or *strongly agreed* with the following statements (based on the Technology Acceptance Model): *Using Ada would enable me to record my medical symptoms and problems quickly* (33/40, 83%), *Learning to use Ada would be easy for me* (37/40, 93%), *I would find it easy to get Ada to do what I want it to do* (26/40, 65%), *The way to use Ada was clear and understandable* (35/40, 88%), *I would find Ada flexible to interact with* (35/40, 88%), *It would be easy for me to become skillful at using Ada* (34/40, 85%), and *I would find Ada easy to use* (36/40, 90%). The mean score for these 7 questions was 84.6%. Free-text comments were prompted by two questions: “In your own words, what was MOST helpful about Ada?” and “In your own words, what was the biggest problem in using Ada?” The results are summarized in Table 6.

**Table 4:** Results of the user survey on previous use of technology and information seeking behavior model (N=40).

Question type, question, and response options			Participants, n (%)	
**Questions on use of technology and looking for medical information**				
	**Question 1: Do you use a cellphone to send SMS text messages?**			
		No	1 (3)	
		Sometimes	4 (10)	
		Often	35 (88)	
	**Question 2: Do you use a cellphone to enter or view information?**			
		No	3 (8)	
		Sometimes	7 (18)	
		Often	30 (75)	
	**Question 3: Do you use a computer at work?**			
		No	19 (48)	
		Sometimes	3 (8)	
		Often	18 (45)	
	**Question 4: Where would you MOST OFTEN look for medical advice?**			
		Doctor (physician)	22 (55)	
		Pharmacist	1 (3)	
		Family	1 (3)	
		Friend	0 (0)	
		On the web	16 (40)	
		Other	0 (0)	
	**Question 5: Do you search for medical symptoms on the web?**			
		No	7 (18)	
		Sometimes	19 (48)	
		Often	14 (35)	
	**Question 6: Have you used a diagnosis program or symptom checker before?**			
		No	29 (73)	
		Sometimes	10 (25)	
		Often	1 (3)	

**Table 5:** Results of the user survey questions derived from the Technology Acceptance Model (N=39).

Question type, question, and response options		Participants, n (%)	
**Question 7: In general, how satisfied were you with Ada?**			
	Very unsatisfied	0 (0)	
	Unsatisfied	12 (31)	
	Neutral	14 (36)	
	Satisfied	13 (33)	
	Very satisfied	0 (0)	
	Top 2	13 (33)	
**Question 8: How enjoyable did you find using Ada?**			
	Very unpleasant	0 (0)	
	Unpleasant	16 (40)	
	Neutral	15 (38)	
	Enjoyable	9 (23)	
	Very enjoyable	0 (0)	
	Top 2	9 (23)	
**Question 9: Were you expecting it to be different than it was?**			
	No	31 (78)	
	Yes	9 (23)	
**Question 10: If a friend were in need of similar help, how likely would you be to recommend Ada to them?**			
	Very unlikely	1 (3)	
	Unlikely	2 (5)	
	Neutral	9 (23)	
	Likely	13 (33)	
	Very likely	15 (38)	
	Top 2	28 (70)	
**Question 11: Using Ada would enable me to record my medical symptoms and problems quickly.**			
	Strongly disagree	1 (3)	
	Disagree	1 (3)	
	Neutral	7 (18)	
	Agree	21 (53)	
	Strongly agree	12 (30)	
	Top 2	33 (83)	
**Question 12: Learning to use Ada would be easy for me.**			
	Strongly disagree	0 (0)	
	Disagree	2 (5)	
	Neutral	1 (3)	
	Agree	24 (60)	
	Strongly agree	13 (33)	
	Top 2	37 (93)	
**Question 13: I would find it easy to get Ada to do what I want it to do.**			
	Strongly disagree	0 (0)	
	Disagree	2 (5)	
	Neutral	12 (30)	
	Agree	20 (50)	
	Strongly agree	6 (15)	
	Top 2	26 (65)	
**Question 14: The way to use Ada was clear and understandable.**			
	Strongly disagree	0 (0)	
	Disagree	2 (5)	
	Neutral	3 (8)	
	Agree	21 (53)	
	Strongly agree	14 (35)	
	Top 2	35 (88)	
**Question 15: I would find Ada flexible to interact with.**			
	Strongly disagree	0 (0)	
	Disagree	0 (0)	
	Neutral	5 (13)	
	Agree	26 (65)	
	Strongly agree	9 (23)	
	Top 2	35 (88)	
**Question 16: It would be easy for me to become skillful at using Ada.**			
	Strongly disagree	0 (0)	
	Disagree	2 (5)	
	Neutral	4 (10)	
	Agree	22 (55)	
	Strongly agree	12 (30)	
	Top 2	34 (85)	
**Question 17: I would find Ada easy to use.**			
	Strongly disagree	0 (0)	
	Disagree	0 (0)	
	Neutral	4 (10)	
	Agree	24 (60)	
	Strongly agree	12 (30)	
	Top 2	36 (90)	

**Table 6:** Summary of free-text comments from the survey (N=40).

Question and responses		Participants, n (%)
**In your own words, what was MOST helpful about Ada?**		
	*Described the system as easy to use or understand* ^a^	14 (35)
	*Referred to good questions or history taking*	16 (40)
	*No data* or said “no comment” or similar	5 (13)
	*Other* comments, including “took mind off pain” and “instant information”	5 (13)
**In your own words, what was the biggest problem in using Ada?**		
	*No comment*	24 (60)
	*Difficulties with using it, mostly expressions of inexperience*, including “don’t like technology/apps,” “inexperience,” “crashed,” and “initially confusing but then fairly simple.”	7 (18)
	*Issues with questions and answers*, including “not enough choices during questions,” “putting in multiple symptoms,” and “I have a lot of symptoms and it was hard to keep track of which one that app was asking more information on.”	6 (15)
	*Other*, including “trying to type with migraine” and “wanted the diagnosis.”	2 (5)

^a^General descriptions of categories italicized.

## Discussion

### Principal Findings

This study provides preliminary data supporting both the feasibility and accuracy of a symptom checker app in an ED setting. To determine whether a symptom checker such as Ada is likely to be beneficial to patients and health systems, it is necessary to determine if it is (1) accurate at diagnosing patients based on their reported symptoms, (2) safe in its triage recommendations without a high level of overdiagnosis, (3) usable by a wide range of patients, and (4) able to positively influence patient decision-making in seeking appropriate care. This observational study provides insights into each of these criteria. The decision to test the Ada app, designed for home or community use, in an ED setting addresses the critical need to understand the performance of such tools for a full range of patient presentations. Determining the performance of a symptom checker in patients who are acutely ill in a community study would require a very large sample size and make the assessment of user experience challenging. This study is part of a 3-step evaluation plan covering different levels of patient acuity: (1) the ED-based study, (2) a similar study being completed in urgent primary care at Brown Medicine in Rhode Island, and (3) planned studies of app usage data in the community.

The overall performance of Ada on its top 5 diagnoses compared with the ED diagnoses was not significantly different from that of the study physicians assessed on their top 3 diagnoses. When compared based on the top 1 diagnoses, the physicians had substantially higher scores, with one being significantly more sensitive than Ada. As the physicians’ diagnoses were based purely on the data collected by Ada, this suggests that the Ada algorithm could be improved in the area of ranking of diagnoses. A similar result was observed in a study of medical students and Ada diagnosing 3 case vignettes in rheumatology. Ada’s performance was lower on the top 1 diagnosis but almost the same on the top 5 [34]. When the Ada diagnoses were critiqued by the physicians, 15% (19/130) were considered *unreasonable*, and <1% (1/130) were considered *very unreasonable*. The percentage of agreement between pairs of physicians on the final diagnoses was higher than their level of agreement with Ada, but a larger study would be required to determine if this was significant.

Scores for *comprehensiveness* and *relevance* were low in this study because of the presence of 3 to 5 diagnoses in the differential list. The relevance of the physicians might increase if they were not required to record 3 diagnoses for each case*.* Overall, the performance of Ada was very similar to the physicians’ mean scores and matched their comprehensiveness if the top 5 diagnoses were included, but patients may have difficulty interpreting 5 options, especially as this means that the most accurate diagnosis could be one of the last two. Of note, Ada’s correct diagnoses were nearly all the same as those of physician 1, suggesting that, where symptom data were adequate, the performance was good. The availability of vital sign data had little effect on the physicians’ differential diagnoses or triage.

At least two independent emergency physicians rated Ada’s triage recommendations as safe in 86% (32/37) of the patients. Although none of the remaining 14% (5/37) of patients whose triage recommendations were scored as *too risky* experienced an adverse outcome in the ED, the study was not powered to detect uncommon or rare serious conditions or provide longer follow-up. When considering how to improve the safety of triage recommendations of symptom checkers, weight should be given not only to the seriousness of the *most likely* diagnoses but also to the *riskiness* of certain clusters of symptoms that may represent a less common but serious condition. Many of the patients studied underwent evaluation to rule out serious conditions such as myocardial infarction. A negative evaluation does not mean that the evaluation and level of care were inherently incorrect. For example, in this study, a diagnosis of AMI by Ada or a physician using the data collected by Ada was considered correct if the patient was screened for AMI in the ED, even if the screen was negative.

We are not aware of a study of patients directly entering data on their own symptoms to evaluate the accuracy of both diagnosis and triage in a general ED population. A study in 2 Canadian EDs and 13 primary care practices evaluated a symptom checker developed by the team that recommended a triage level out of 4 options [35]. For 281 hospital patients, the sensitivity for emergencies was 10/10 (100%) and, for urgent cases, it was 73/81 (90%), but performance for routine and home care was poorer at 52% and 29%, respectively. The positive predictive value was 40% for allocation to the hospital, 93% for primary care, and 32% for home care. They had to exclude 50% of the patients because of lack of access to the visit record and 22% of the remainder as the patients entered a different presenting complaint. The triage performance of Ada and other symptom checkers can be compared with telephone or nurse-based triage systems. A report showed a median accuracy of 75% and potential harm from undertriage of 1.3% to 3.2% [36], although there was wide variation in the reported performance in that review. Tam et al [37] reviewed a range of studies of triage in the ED and primary care in 2018, including designs using case vignettes or retrospective chart reviews. They reported that “when comparing with all multi-center studies, both methods revealed a triage accuracy of about 60% and about 23% of cases [it] was under-estimated,” although some single-site studies had higher accuracies of >70%.

The user survey showed that the patients had a wide range of ages (up to 76 years, with 12/40, 30% being aged ≥50 years); a broad range of levels of schooling; and a wide range of racial, ethnic, and socioeconomic backgrounds (Table 1). Although they had mixed views on their overall satisfaction with Ada, this was likely their first use of the app and, for 73% (29/40), their first use of any symptom checker. They had generally positive views on the system and would recommend it to others. This was in the context of patients typically feeling very unwell and in a stressful ED environment, providing some confidence that patients can use a well-designed symptom checker even when sick. Satisfaction scores would presumably be higher if the patients had been given access to their diagnoses and triage results, as noted in some patient comments (eg, “wanted the diagnosis”).

A previous study of Ada that assessed its ease of use in a primary care setting in the United Kingdom [28] showed that younger patients were more likely to report that Ada provided helpful advice, but there was no effect seen from patients’ sex. A study in California [18] also examined the mix of patients using Ada in a health system and found that the patient characteristics of users were similar to those of their general patient population but with a younger mean age. A study by Knitza et al [38] on the use of Ada, a custom diagnostic app (Rheport), and web-based searching for symptoms, showed a high System Usability Scale score of 77.1 for Rheport and a somewhat lower score for Ada of 74.4 (*P*<.001). Ratings for “very helpful” or “helpful” were higher for Rheport (65.8% vs 44.3%), although similar numbers would recommend each system to others (79.2% vs 73.3%).

### Limitations

Some technical problems occurred with the research environment for Ada developed for the study (not with the public-facing production system), which led to missing or incomplete reports in some cases. These problems were addressed in the follow-up study.

The greatest challenge encountered in this study design was defining the “correct diagnosis” based on the clinicians’ assessment and EHR notes from the ED visit. In many cases, the ED physicians’ role was to exclude serious causes for the presenting symptoms, with the patient potentially seeking investigation through their primary care physician or specialist at a later date. The multiple possible diagnoses recorded in the ED physicians’ notes also make it difficult to compare metrics on matching diagnoses with many previous studies of symptom checkers. In the studies by Semigran et al [12] and Gilbert et al [13], clinical vignettes had just 1 correct diagnosis, which, as shown here, is not typical for actual patient assessments in the ED. The *comprehensiveness* and *relevance* metrics consider the full set of diagnoses in each differential. In the large vignette study by Gilbert et al [13], *comprehensiveness* for Ada was similar to that in this study (48% on the top 3 diagnoses vs 41% in this study), but relevance was higher (45% on the top 3 diagnoses vs 22% in this study); Ada’s performance improved in this study when the top 5 diagnoses were included.

In addition, in those cases where a comparison was able to be made with the ED physicians’ diagnoses, >27% (8/30) were not correctly diagnosed by either Ada or the study physicians. This is likely due in part to the lack of data on physical examinations and investigations available to the ED physician. Berry et al [39] compared the diagnostic accuracy of (1) physicians reviewing the symptom data items collected by the symptom checker (similar to this study) with (2) adding the clinical data collected by the reviewing physician. They confirmed that the additional clinical data significantly improved diagnostic accuracy. The use of the symptom data from Ada potentially limits the diagnostic accuracy of the physician reviewers as they cannot ask additional questions. This may lead to underestimating the physician performance compared with Ada’s.

We collected data in 2019 before the COVID-19 pandemic and, therefore, did not include patients with COVID-19. Initial studies of symptom checkers on clinical case descriptions of patients with COVID-19 have shown a wide range of performance for different symptom checkers on different data sets. The follow-up study is expected to include some patients with COVID-19.

### Comparison With Other Studies

Semigran et al [12] studied 23 symptom checkers using 45 clinical vignettes. The best-performing symptom checkers had a sensitivity for the correct diagnosis for their top 1 diagnosis in 35.5% of cases and for the top 3 diagnoses in 51% of cases, compared with independent physicians, who achieved 72% top-1 sensitivity and 84.3% top-3 sensitivity [23]. The 3 best-performing systems were closer in performance to the physicians, with top-1 sensitivity of 43% to 50% and top-3 sensitivity of 67% to 71%. A recent repeat study used the vignettes by Semigran et al [12] and versions of symptom checker apps available in 2020 [40]. Top-1 sensitivity was 10% higher at 45.5%, and top-10 sensitivity was 71.1% (compared with 55.8% for the top 20 diagnoses in the study by Semigran et al [12]). Top-1 sensitivity for Ada was higher than the average at 53%, with the top-10 sensitivity average at 71%. Another study that sought to replicate the study by Semigran et al [12] was published by Hill et al [24] in 2020 with symptom checker apps available in Australia. In that study, the mean sensitivity for the correct diagnoses was 36% for the top 1, 52% for the top 3, and 58% for the top 10. In our study, sensitivity based on the top 3 diagnoses was 63.3% for Ada versus 70% for physicians, with a larger difference including only the top 1 diagnoses. However, the results in our study are not completely comparable given that many cases had more than one ED diagnosis (potentially increasing apparent performance) and were based on data entry by patients of their own symptoms (potentially decreasing performance). A recent study on the use of Ada for mental health disorders by Henneman et al [29] used a similar study design and metric to our RIH study, with 49 patients (61% female) using the app before seeing a psychotherapist. As in our RIH study, they used the metric for a correct diagnosis as *at least one diagnosis from the Ada differential diagnosis matching with one from the psychotherapist*. They showed a correct match (sensitivity) of the Ada top 1 diagnosis in 51% (95% CI 37.5%-64.4%) of cases and with Ada’s top 5 diagnoses in 69% (95% CI 55.4%-80.6%) of cases, a higher performance than in our RIH study for the top 1 diagnosis but equivalent for the top 5 diagnoses. The interrater reliability varied widely depending on the condition.

In a systematic review, Chambers et al [7] included all available evaluation studies of symptom checkers used for the assessment of urgent conditions up to April 2018. A total of 27 studies were included in the final review. They identified the potential risks associated with symptom checker use as “increasing demand,” “duplicating healthcare contacts,” and “providing advice that is not safe or clinically appropriate.” They found “little evidence to indicate whether or not digital and online symptom checkers are detrimental to patient safety.” Limitations in the 6 studies that measured safety included being short-term; having small samples and, therefore, including insufficient adverse events; and being limited to specific symptoms or from specific populations unrepresentative of urgent care users. Among the priorities for research, they identified qualitative research to investigate perceptions of symptom checkers and barriers to their use by people who are less familiar with digital technology. Our study provides a model for the assessment of safety and usability based on direct use by patients with serious and potentially life-threatening conditions, but this sample size does not allow for a clear assessment of triage accuracy and safety. In addition, an intervention study would be required to show whether changes in patients’ decisions to seek urgent care based on the symptom checker output affected the quality and safety of care. More recent studies by our group have a larger sample size, and planned studies will address the impact on patient decision-making.

Previous studies of symptom checkers in urgent or ED settings have generally been limited by the selection of less urgent patients—for example, Mediktor [41]—or lack of direct use of symptom checkers by patients. Berry et al [39] studied WebMD, iTriage, and FreeMD symptom checkers for the diagnosis of cough in primary care. The best-performing symptom checker had a sensitivity for the correct diagnosis of 34.5% (top 1 diagnosis) and 71.6% (top 3 diagnoses). However, the symptom data came from paper forms patients filled out in the waiting room, not from direct use of an app; moreover, the study had a small list of possible diagnoses, and the “correct diagnosis” was allocated by one primary care physician, limiting the potential generalization of the findings. A team at the Queen Mary Hospital in Hong Kong studied the triage accuracy of 2 symptom checkers based on a random sample of 100 charts from the ED [42]. Triage accuracy was rated as low at 74% and 50%, with poorer performance on more urgent cases; diagnostic accuracy was not tested. A recent study showed that patients reading simplified case vignettes based on the study by Semigran et al [12] had better triage performance than symptom checkers on low-risk conditions but poorer performance in detecting cases requiring urgent care [43]. This suggests that there may be an important role for symptom checkers in identifying serious conditions missed by patients, but that only the best-performing symptom checkers are likely to be effective.

Developments in symptom checker algorithms in the last 7 years would have been expected to improve triage performance (in line with improvements in diagnosis) but, as shown by Schmieding et al [40], there are still many symptom checkers that are less safe and more prone to overtriage and possibly undertriage than patients themselves. In the aforementioned 2020 study by Schmieding et al [40] using the protocol and vignettes by Semigran et al [12] (with small modifications), the median triage accuracy was 55.8%, similar to the 2015 study by Semigran et al [12] (59.1%), but the ratio of over- to undertriage shifted, with overtriage only slightly higher than undertriage. This led to the apps missing >40% of the emergency vignettes overall, a real concern for patient safety. The Ada app performed better than average with a triage accuracy of 64%, which is close to the results of our RIH study, and a high accuracy for triage of emergencies (89%). The study by Hill et al [24], which also replicated the study by Semigran et al [12], showed a mean triage accuracy of 49%, with stronger performance on emergency cases. The study by Chan et al [35] (reported earlier) showed significantly better triage accuracy by their locally developed symptom checker than the patients overall (73% vs 58%; *P*<.01), and performance on emergency and urgent cases was stronger than on routine or home care. A recent study on the use of Ada by 378 “walk-in” patients in urgent care compared its triage accuracy with the result of a triage nurse using the Manchester Triage System [44]. The app was shown to undertriage 8.9% of cases and overtriage 57.1%, although physician assessment of the undertriaged cases suggested that 14 cases (3.7%) did not represent a risk of an adverse event. Overtriage was significantly higher than that in our RIH study. The results are not fully comparable as all triage performances in our RIH study were judged by 3 physicians rather than by a triage nurse.

### Potential Change in Patient Decision-making

There is initial evidence that patients may change the urgency level of the care they seek based on the results of symptom checkers and other web-based diagnostic tools. A study of 158,000 patient consultations with the symptom checker Buoy evaluated the intended urgency level of the care they would seek before and after viewing the output of the symptom checker. A total of 32% of patients stated that they would seek a lower urgency level, and 4% would seek a higher level [26]. A study of Ada in use by patients in a primary care practice in the United Kingdom evaluated any change in their intended acuity of care based on the diagnosis and triage results. A total of 12.8% of the patients stated that they would seek less urgent care, and 1.2% said that they would seek more urgent care [28]. Therefore, it is likely that a proportion of patients using symptom checkers will change their care-seeking behavior based on the results presented. The main limitation of these 2 studies is that there is no indication of whether the advice received was accurate or safe. Some evidence of the benefits of web-based health data was shown in a study of 5000 laypeople who were asked to review clinical vignettes of different illnesses and then provide their assessment of the appropriate triage level and top 3 diagnoses [45]. After viewing web-based information about the case (most commonly search engines and specialist medical sites), the participants’ diagnostic accuracy improved modestly from 49.8% to 54% (*P*<.001). There was no change in triage accuracy. A similar study by Martin et al [46] compared the ability of patients with low-acuity symptoms in an ED to match at least two of the differential diagnoses made by the physician who subsequently assessed them. A total of 300 patients were randomized to (1) receive assistance from a standard Google search, (2) receive assistance from a Google search with enhanced medical features, or (3) have no access to searching. There was no significant difference in the percentage matching 2 physician diagnoses (27%, 28.3%, and 23.8%, respectively). Given the potential for patients to seek less urgent care based on symptom checker assessment results and the limited evidence of improved diagnostic accuracy from widely used search engines, studies of diagnosis and triage accuracy of symptom checkers are essential. In addition, studies are required on the effects of symptom checker output on patients’ care-seeking behavior and the safety and appropriateness of those decisions.

### Conclusions

The primary goal of this study was to answer whether a widely used symptom checker was safe, effective, and usable by patients who were acutely ill and might have a life-threatening disease. These data, in the context of existing studies of symptom checker apps, including Ada, should help provide validation of diagnosis and triage accuracy with real patient use. This pilot study provides evidence to support usability and on overall diagnostic performance while showing the potential for improving the ranking of diagnoses by Ada. On triage, performance was similar to that of the clinicians in most cases but with significant overtriage and some undertriage. A larger study would be required to provide definitive evidence, and assess the potential impacts on care-seeking behavior. The results also demonstrate a fundamental challenge in developing and evaluating such systems—gaps and variability in the documentation of differential diagnoses in EHRs. A larger study is underway of patients requesting urgent primary care appointments, with patients completing the consent form, the Ada questions, and the user survey in the home or community setting. In addition to broadening the range of diagnoses and patient types, this will allow for a better assessment of appropriate triage levels. The goal is also to use the research environment and data to study a range of symptom checkers. The design of this study should provide a model for larger and more varied evaluation studies of real-world performance and use of symptom checkers. Good performance in observational studies of this sort is a requirement for measuring the likely clinical impact in intervention studies, including randomized controlled trials.

## Appendix

Multimedia Appendix 1 Example Ada case output.

Multimedia Appendix 2 Table of all study cases, including presenting complaints and emergency department diagnoses.

Multimedia Appendix 3 Diagnoses in cases where Ada urgency or triage advice was rated as unsafe or too risky.

## Abbreviations

AMI: acute myocardial infarction
ED: emergency department
EHR: electronic health record
REDCap: Research Electronic Data Capture
RIH: Rhode Island Hospital
