# Supplementary material for: Evaluation of Diagnostic and Triage Accuracy and Usability of a Symptom Checker in an Emergency Department: Observational Study
Source: JMIR Mhealth Uhealth. 2022 Sep 19;10(9):e38364. doi: 10.2196/38364 (PMC9531004; doi:10.2196/38364)
Supplement: Multimedia Appendix 1 [file mhealth_v10i9e38364_app1.pdf]

Assessment Report

General Information

Advice Level

Callambulance

Patient

Age: 60
Sex: Male
Language: EN-GB

Presenting Complaint(s)

Dizziness
Chest pain
Loss of sensation in the arm
Nausea

Reported Symptoms and Factors *(F = Factors)*

Reported as present

- Smoker (F)
- Diabetes (F)
- Dizziness
  - Time since onset: less than one day
  - Standing up: worsens
  - Type of dizziness: like a feeling of lightheadedness
- Chest pain
  - Time since onset: one day to one week
  - Location: behind the breastbone
  - Intensity: mild
  - Radiating: no
  - Tender to the touch: no
  - Breathing: no effect
- Loss of sensation in the arm
  - Time since onset: less than one day
  - Distribution: one side
- Nausea
  - Time since onset: less than one day
  - Intensity: mild
- Chest tightness
  - Physical activity: no effect
- Diarrhoea
  - Bloody diarrhoea: no
  - Watery diarrhoea: no
- Shoulder pain

Reported as absent

- High blood pressure (F)
- Abnormal heartbeat sensation
- Difficulty breathing
- Sudden intense fear
- Cold skin on arms or legs
- Loss of consciousness
- Rapid pulse
- Fall
- Abdominal pain
- Loss of appetite
- Pins and needles in the lower leg
- Vomiting
- Headache
- Restlessness
- Increased tendency to sweat
- Fast breathing
- Pale face
- Weakness with everyday tasks or play

Reported as uncertain

- Activity: worsens
- Laterality: one side
- Intensity: mild

Possible Causes

1 Heart attack

CallAmbulance

4 out of 10 people with these symptoms had this condition.

Associated Present Findings

Smoker, Diabetes, Dizziness, Chest pain, Nausea, Chest tightness, Diarrhoea, Shoulder pain.

Associated Absent Findings

High blood pressure, Abnormal heartbeat sensation, Difficulty breathing, Sudden intense fear, Cold skin on arms or legs, Loss of consciousness, Rapid pulse, Fall, Abdominal pain, Loss of appetite, Vomiting, Headache, Restlessness, Increased tendency to sweat, Fast breathing, Pale face, Weakness with everyday tasks or play.

2 Unstable angina pectoris

CallAmbulance

3 out of 10 people with these symptoms had this condition.

Associated Present Findings

Smoker, Diabetes, Dizziness, Chest pain, Nausea, Chest tightness, Shoulder pain.

Associated Absent Findings

High blood pressure, Abnormal heartbeat sensation, Difficulty breathing, Sudden intense fear, Cold skin on arms or legs, Loss of consciousness, Rapid pulse, Fall, Abdominal pain, Loss of appetite, Vomiting, Restlessness, Increased tendency to sweat, Fast breathing, Pale face, Weakness with everyday tasks or play.

3 Acute panic attack

PrimaryCare4Hours

1 out of 100 people with these symptoms had this condition.

Associated Present Findings

Dizziness, Chest pain, Nausea, Chest tightness, Diarrhoea.

Associated Absent Findings

Abnormal heartbeat sensation, Difficulty breathing, Sudden intense fear, Loss of consciousness, Rapid pulse, Fall, Abdominal pain, Pins and needles in the lower leg, Vomiting, Headache, Restlessness, Increased tendency to sweat, Fast breathing, Pale face.

4 Atrial fibrillation

CallAmbulance

1 out of 100 people with these symptoms had this condition.

Associated Present Findings

Diabetes, Dizziness, Chest pain, Chest tightness.

Associated Absent Findings

High blood pressure, Abnormal heartbeat sensation, Difficulty breathing, Sudden intense fear, Cold skin on arms or legs, Loss of consciousness, Rapid pulse, Fall, Restlessness, Increased tendency to sweat, Fast breathing, Pale face, Weakness with everyday tasks or play.
