# Supplementary material for: Evaluation of Diagnostic and Triage Accuracy and Usability of a Symptom Checker in an Emergency Department: Observational Study
Source: JMIR Mhealth Uhealth. 2022 Sep 19;10(9):e38364. doi: 10.2196/38364 (PMC9531004; doi:10.2196/38364)
Supplement: Multimedia Appendix 3 [file mhealth_v10i9e38364_app3.docx]

**Examples of cases where one or more physicians assessed Ada triage levels as "unsafe and too risky"**

Yellow highlights: cases where diagnoses 2 – 5 included a triage level more acute than the overall triage level, and which would likely reduce number of cases where triage was assessed as unsafe and too risky.

| **Case #** | **Number of reviewers** | **Assessor** | **Triage** | **Diagnoses** |
| --- | --- | --- | --- | --- |
|  |  |  |  |  |
| **Case 2** |  | ED |  | Dizziness, no final ED diagnosis |
|  |  | Ada | Selfcare pharma | Dx 3 – primary care 4 hours |
|  | 2 | MD | Go to ED | Sepsis, dehydration, arrhythmia |
|  |  | MD | See GP same day | Migraine, Flu-like illness, Heat exhaustion |
|  |  |  |  |  |
| **Case 9** |  | ED |  | Hypokalemia |
|  | 1 | Ada | Primary care 2 To 3 Days | Two at this level, one less acute |
|  |  | MD | See GP same day | Hypothyroidism, Primary mood disorder, Inflammatory bowel disease |
|  |  |  |  |  |
| **Case 10** |  | ED |  | LUQ Abdominal pain, breathless, no diagnosis |
|  |  | Ada | prim. Care 4hr | Dx 2 has call ambulance |
|  | 2 | MD | Go to ED | Panic attack, colitis, arrhythmia |
|  |  | MD | Go to ED | Panic attack, Anaphylaxis, Cardiac arrhythmia |
|  |  |  |  |  |
| **Case 12** |  | ED |  | MI Screen, Acute hyperventilation syndrome, Paresthesias, R Wrist pain,  Type 2 DM w/ hyperglycemia, w/out long-term current use of insulin, Muscle spasm of back, |
|  |  |  |  |  |
|  |  | Ada | Prim. Care 2-3 days | Same level all 3 |
|  | 2 | MD | Go to ED | Anxiety, radiculopathy, electrolyte abnormality [less likely but in list: PE, ACS...] |
|  |  | MD | See GP same day | Stable angina, Asthma, Congestive heart failure |
|  |  |  |  |  |
| **Case 15** |  |  |  | Migraine, temporal arteritis |
|  |  | Ada | Primary care same day | Diagnosis 3: primary care 4 hours |
|  | 1 | MD | Go to ED | Temporal arteritis, Migraine, Acute angle closure glaucoma |
|  |  |  |  |  |
| **Case 19** |  |  |  | E coli infection, Pyelonephritis |
|  |  | Ada | Primary care 4 Hours | All 3 diagnoses same level |
|  | 1 | MD | ED | PID, tubo-ovarian abscess, pyelonephritis |
|  |  |  |  |  |
| **Case 34** |  | ED |  | Hemorrhoids, rectal bleeding |
|  |  | Ada | prim. care 2-3 days | Diagnosis 2 primary care same day, diagnosis 3 emergency care |
|  | 3 | MD | ED | Anal fissure, colitis, cancer |
|  |  | MD | GP same day | Anal fissure, Inflammatory bowel disease, Perianal abscess |
|  |  | MD | ED | Colitis, anal fissure, peri-rectal abscess |
|  |  |  |  |  |

| **Case 38** |  | ED |  | Bacterial vaginosis, STI |
| --- | --- | --- | --- | --- |
|  |  | Ada | Primary care 2 To 3 Weeks | All 5 diagnoses at same triage level |
|  | 3 | MD | See GP same day | Dysmenorrhea, ectopic pregnancy , PID |
|  |  | MD | See GP same day | Dysmenorrhea, Endometriosis, Ruptured ovarian cyst |
|  |  | MD | Go to ED | Ectopic pregnancy, dysfunctional uterine bleeding, menorrhagia |
|  |  |  |  |  |

**A) Rated unsafe and too risky based on top 1 Ada triage level**

| **Total cases** | 37 |  |
| --- | --- | --- |
| **1+ physician** | 8 | 22% |
| **2+ physicians** | 5 | 14% |
| **3 physicians** | 2 | 5% |

**B) Rated unsafe and too risky based on most acute Ada triage levels**

| **Total cases** | 37 |  |
| --- | --- | --- |
| **1+ physician** | 6 | 16% |
| **2+ physicians** | 2 | 5% |
| **3 physicians** | 1 | 3% |
